# Exploiting Plant Volatile Organic Compounds (VOCs) in Agriculture to Improve Sustainable Defense Strategies and Productivity of Crops

**DOI:** 10.3389/fpls.2019.00264

**Published:** 2019-03-19

**Authors:** Federico Brilli, Francesco Loreto, Ivan Baccelli

**Affiliations:** ^1^ Institute for Sustainable Plant Protection, National Research Council of Italy, Florence, Italy; ^2^ Department of Biology, Agriculture and Food Sciences, National Research Council of Italy, Rome, Italy

**Keywords:** volatile organic compounds, defense priming, abiotic and biotic stresses, sustainable crop production, smart agriculture

## Abstract

There is an urgent need for new sustainable solutions to support agriculture in facing current environmental challenges. In particular, intensification of productivity and food security needs require sustainable exploitation of natural resources and metabolites. Here, we bring the attention to the agronomic potential of volatile organic compounds (VOCs) emitted from leaves, as a natural and eco-friendly solution to defend plants from stresses and to enhance crop production. To date, application of VOCs is often limited to fight herbivores. Here we argue that potential applications of VOCs are much wider, as they can also protect from pathogens and environmental stresses. VOCs prime plant’s defense mechanisms for an enhanced resistance/tolerance to the upcoming stress, quench reactive oxygen species (ROS), have potent antimicrobial as well as allelopathic effects, and might be important in regulating plant growth, development, and senescence through interactions with plant hormones. Current limits and drawbacks that may hamper the use of VOCs in open field are analyzed, and solutions for a better exploitation of VOCs in future sustainable agriculture are envisioned.

## Is Conventional Agriculture Ready for a New Challenge?

Reports analyzing the status of agriculture worldwide forewarn that a significant increase of the present agricultural production would be necessary to meet the future demand for food, as the world population is expected to rise from 7.3 to 9.7 billion by 2050. As a consequence, FAO recently projected that an increase of food production (70%) may be required and thus a third green revolution needs to be attained (FAO, 2017). Enhanced crop yields in the past 50 years were made possible by the introduction of mechanization, the progresses in genetics and the use of improved crop varieties, as well as the extensive use of chemicals such as fertilizers and pesticides. However, the yield of grain crops considered the main sources of human and livestock calories (e.g., rice) have already reached a “plateau” (Grassini et al., 2013). Natural resources providing fertilizers (especially phosphate, Peñuelas et al., 2013) are also depleting, and the use of chemicals has caused serious problems with food safety and environmental pollution (Conley et al., 2009). Moreover, climate change is forecasted to increase the severity and frequency of drought events (IPCC, 2014) that will cause both a reduction in plant primary production (Zhao and Running, 2009) and a future progressive exposure of agricultural soils to degradation and loss of fertility (Köberl et al., 2011). Climate change will also favor the spreading of plant pathogens into larger geographical areas where new hosts may be found (Baker et al., 2000), leading to more frequent epidemics (Anderson et al., 2004). In this context, agriculture is called to provide solutions to increase yields while preserving natural resources and the environment.

This perspective article explores the potential of natural Volatile Organic Compounds (VOCs) emitted by plants as an eco-sustainable strategy to implement future smart agricultural practices and enhance plant protection and productivity. As airborne signals, VOCs allow quick defense signaling between distant plant organs (Heil and Silva Bueno, 2007) and the communication between plants (Baldwin et al., 2006). In addition, VOCs can “prime” the defense system of plants for an enhanced resistance to an upcoming stress (Conrath et al., 2002). However, to date, VOCs have been mostly applied in the field to repel herbivores or to attract herbivores’ parasitoids or predators (De Moraes et al., 1998; Dicke and Baldwin, 2010).

## Volatile Organic Compounds (VOCs) Can be a Natural and Eco-Friendly Solution to Enhance Both Crop Defense and Production

### VOCs Protect and “Prime” Plants to Withstand Biotic and Abiotic Stresses

**Figure 1:**
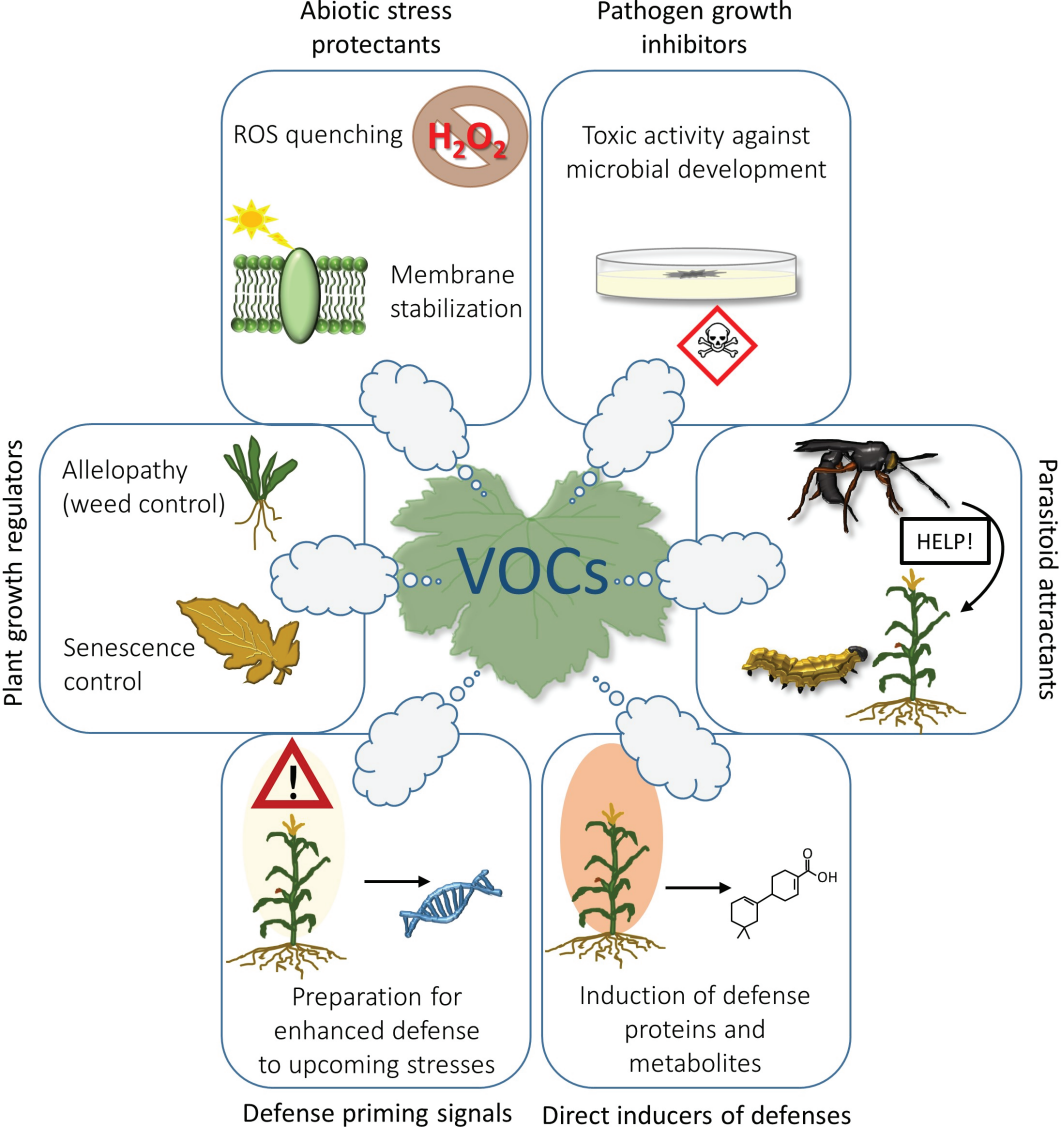
Possible applications of plant VOCs in agriculture: isoprenoids emitted by leaves can exert a protective effect against abiotic stressors by quenching ROS or by strengthening the cell membranes; some VOCs are able to inhibit germination and growth of plant pathogens *in vitro*; herbivore repellency and attraction of herbivore’s parasitoids on infested plants are probably the most known capacity of VOCs; VOCs can impact on plant defensive system by inducing the synthesis of defense proteins and metabolites (e.g., phytoalexins) that impair microbial colonization; VOCs can also act as priming stimuli by inducing epigenetic changes and accumulation of transcription factors that may facilitate faster expression of plant defenses (a DNA helix is thus shown in the figure), thereby enhancing tolerance or resistance to a future stress episode; VOCs can interact with the mechanism of senescence, or be exploited to fight against unwanted weed species (allelopathic effects).

Emission of VOCs can be induced at any time from leaves of all plant species following abiotic (Loreto et al., 2006; Loreto and Schnitzler, 2010) or biotic stresses (Dicke and Baldwin, 2010). Results from many studies have demonstrated that emission of isoprenoids, the most abundant group of VOCs (Guenther et al., 2006), is stimulated by abiotic stresses and improves plant resistance either by direct quenching of reactive oxygen species (ROS) (Loreto and Velikova, 2001), or indirectly by stabilizing cell membranes (Velikova et al., 2011). However, protection of cell membranes to avoid toxic accumulation of ROS is only one among the many roles of VOCs that may be exploited in agriculture (Figure 1).

Plants maintain memory of any stress event they have experienced (Crisp et al., 2016; Hilker et al., 2016), and this memory is able to influence the response to forthcoming stressful situations. Factors able to shape the plant’s stress memory are referred to as “priming stimuli”, among which plant VOCs play a crucial role because, due to their volatility, they can quickly reach distant plant parts (Heil and Kost, 2006; Mauch-Mani et al., 2017). A “primed” plant shows an earlier, stronger, and faster response upon further stress occurrence, thereby resulting in increased resistance and/or tolerance (Conrath et al., 2015; Mauch-Mani et al., 2017). VOCs have been extensively demonstrated to prime defenses against herbivorous insects (Kim and Felton, 2013), pathogens (Ameye et al., 2015), and environmental stresses (Cofer et al., 2018). Defense priming against pathogens has also been considered as a sort of “green vaccination” (Luna-Diez, 2016). Green leaf volatiles (GLVs) such as Z-3-hexenyl acetate, ubiquitously and rapidly released after mechanical damage of leaf tissues (Brilli et al., 2011), have been reported to prime resistance of wheat plants to the fungal pathogen *F. graminearum* (Ameye et al., 2015) and to reduce the damage occurring to maize plants during cold stress (Cofer et al., 2018). Other VOCs such as methyl salicylate (MeSA) and monoterpenes (i.e., camphene and pinene) (Riedlmeier et al., 2017) have been found to actively participate in the mechanisms leading to systemic acquired resistance (SAR) (Dempsey and Klessig, 2012). Low concentrations of methyl jasmonate (MeJA) have been demonstrated to prime plant defenses by modifying the epigenetic status of wound-inducible genes in rice, thereby enhancing responsiveness to wounding (Bertini et al., 2018). Even methanol, ubiquitously emitted from plant leaves during cell division and cell wall expansion (Nemecek-Marshall et al., 1995), seems to act as a priming stimulus when released from damaged tobacco leaves by enhancing resistance to the pathogenic bacterium *Ralstonia solanacearum* (Dorokhov et al., 2012). In addition, antibacterial defenses have also been reported to be primed by VOCs such as nonanal in lima bean plants treated with benzothiadiazole (BTH), a synthetic salicylic acid analog (Yi et al., 2009). Compared to the direct induction of defenses in plants, priming does not incur in an energetically costly activation of metabolic pathways (van Hulten et al., 2006; Martinez-Medina et al., 2016) and therefore represents a sustainable method to develop novel crop protection strategies.

### VOCs Inhibit Growth and Development of Plant Pathogens

A number of experimental trials have shown the capacity of various VOCs produced by leaves to inhibit germination and growth of plant pathogens, yet the mechanisms of action remain unknown. Citral, carvacrol, and trans-2-hexenal were reported to be effective in hampering *in vitro* growth and germination of *Monilinia laxa*, the agent of brown rot of stone fruit (Neri et al., 2007). In particular, trans-2-hexenal provided protection also when tested *in vivo* on apricot, nectarine, and peach fruits as a postharvest biofumigant (Neri et al., 2007). In addition, the growth of *Colletotrichum acutatum*, causing citrus post-bloom fruit drop, was moderately inhibited *in vitro* when exposed to linalool (Marques et al., 2014). *Botrytis cinerea*, a necrotrophic fungus with a very broad host range, has been reported to be highly sensitive to the *in vitro* application of monoterpenes, such as (+)-limonene (Simas et al., 2017). However, exposure to (+)-limonene stimulated *in vitro* growth of the fungal pathogen *Penicillium digitatum*, whereas this fungus was highly inhibited by the application of citral (Simas et al., 2017). In a recent work, Quintana-Rodriguez et al. (2018) performed a screening on the efficacy of 22 different VOCs, known to be emitted from leaves, against the fungal pathogens *Colletotrichum lindemuthianum*, *Fusarium oxysporum*, and *B. cinerea*. These fungi were grown in Petri dishes in which the headspace had been enriched, each time, with a single VOC. Results showed that exposure to nonanal, (+)-carvone, citral, *trans*-2-decenal, *L*-linalool, nerolidol, or eugenol significantly inhibited the growth of all these three fungal species, with eugenol demonstrating the strongest activity. Other VOCs such as cuminaldehyde and *p*-cymene have been also demonstrated to possess antifungal activity against *B. cinerea, F. oxysporum*, *Verticillium dahliae*, and *Alternaria mali* (Sekine et al., 2007).

### VOCs Improve Plant Growth and Productivity

Limited information exists concerning the effects of plant VOCs on crop productivity. Emission of VOCs from leaves can have allelopathic effects and impair the growth of other competitive plant species (Arimura et al., 2010). Hexenal (Gardener et al., 1990) and isoprenoids (mono- and sesqui-terpenes) (Fischer, 1986), for instance, have been demonstrated to inhibit seed germination and root growth (Nishida et al., 2005). By mediating competition between plant species, VOCs may allow to control weeds and thus enhance crop productivity through a more efficient acquisition of nutrients, water, and light (Puig et al., 2018).

Moreover, it was recently proposed that VOCs (i.e., isoprenoids) may work in synergy with other secondary metabolites (i.e., carotenoids) and hormones (i.e., cytokinins) which are all synthesized by the methyl erythritol phosphate (MEP) pathway to regulate senescence (Dani et al., 2016). Aging of plant tissues is controlled by changes in hormone levels (Woo et al., 2013) and may lead to uncontrolled accumulation of ROS following damage to cellular organs (i.e., membranes) (Stark, 2005) and other macromolecules (i.e., DNA) (Campisi and Vijg, 2009). Therefore, a sustained production of volatile isoprenoids may synergize with the biosynthesis of cytokinins and increase antioxidant activity at the foliar level. This could prevent cell degradation and death, thus prolonging the life span of leaves and flowers with a positive impact on the whole plant production process.

The trade-off between benefits and costs of VOC emission as stress relief compounds is not clear and difficult to assess. Experiments with transgenic plants suggest that the metabolic cost for emitting isoprene (the most abundant VOC released from leaves) outweighs benefits (Behnke et al., 2012).

## Limitations and Drawbacks to the Application of VOCs in the Field

### Knowledge Boundaries

Despite the demonstrated efficacy of VOCs in enhancing plant fitness and defense against stress in many controlled trials in the laboratory, their effectiveness to enhance plant defenses have been tested in the field only in a few cases (James and Price, 2004; Sugimoto et al., 2014). To date, VOCs are applied in agriculture solely for the “push-pull strategy”, where the crop of interest is both intercropped with plant species that emit VOCs able to repel (“push”) herbivores, and surrounded with plants emitting VOCs that simultaneously attract (“pull”) herbivores away from the field (Stenberg et al., 2015; Picket and Khan, 2016) (Figure 1).

Why are VOCs not more intensively used in agriculture for integrated and eco-friendly plant protection? One reason could be that laboratory experiments demonstrating VOC efficacy have been performed with concentrations far higher than those achievable in open field. Trials to test the antimicrobial activity of VOCs are often performed in Petri dishes by applying pure liquid solution, without even quantifying the real VOC concentration present in the headspace over the course of the experiment. Moreover, while the high biodegradability of VOCs may minimize both their impact and the occurrence of long-term non-targeted effects, at the same time this limits VOC persistence and activity (Glare et al., 2012). Indeed, VOCs are often very reactive with the environment. Reports have demonstrated that VOCs disappear more rapidly in polluted environments where they can react with NOx, OH− radicals, and ozone (McFrederick et al., 2009; Blande et al., 2014). Therefore, high reactivity of VOCs limits the distance they travel across the fields and their overall range of protection on crops. Reduction of VOC concentration in air may thus modulate their effect on insects, microbes, and plants. Finally, the very essence of VOCs (volatility) makes these compounds highly influenced by meteorological factors such as wind speed and direction, humidity and rain, and temperature, among others. It is also important to highlight that VOC emissions from leaves in the field can hardly be faithfully reproduced (Aksenov et al., 2014). In fact, complex blends rather than single VOCs are released by plants, and their biosynthesis is controlled by a network of different biochemical pathways (Dicke et al., 2009; Hare, 2010). In addition, the composition of VOC mixtures is subjected to strong plant genotypic and phenotypic plasticity and can vary according to the plant ontogenetic state (Bracho-Nunez et al., 2011) and environmental conditions (Brilli et al., 2016). Modern techniques of micro- and nano-encapsulation, which allow a more controlled release of synthetic blends that mimic the natural release of VOCs, are expected to improve VOC efficacy (Cabral Marques, 2010). Further efforts should be also made to decipher the perception mechanism of VOCs within plant tissues, since it is not yet clear how VOCs are perceived by plants. Indeed, molecules acting as VOC receptors have not been identified so far.

### Unexpected Results of VOC Application in the Field

Some studies have reported other possible pitfalls of the application of VOCs in open fields that need to be carefully evaluated. For instance, the release of synthetic GLVs in the field synergistically affected isoprenoid emission from maize plants and unexpectedly increased herbivore damage without significantly attracting beneficial insects (von Mérey et al., 2011). Methanol emitted by wounded tobacco leaves was reported to enhance antibacterial defenses in neighboring non-wounded plants, but also facilitated the spreading of tobacco mosaic virus (TMV) (Dorokhov et al., 2012). In other cases, VOC phyto-toxicity was observed. For example, Neri et al. (2007) demonstrated that the concentration of *trans*-2-hexenal required to exert the antimicrobial activity was phytotoxic for some fruits (i.e., apricot, peach, and nectarine), whereas plum fruits could be protected without damage to plant tissues. Importantly, application of Z-3-hexenyl acetate enhanced resistance of wheat plants against the pathogenic fungus *F. graminearum* while boosting the production of deoxynivalenol (DON), a dangerous mycotoxin for human health (Ameye et al., 2015).

### High “Price” to Pay for the Use of VOCs in Agriculture

Technical limits and drawbacks may not have been the only constraints limiting the exploitation of VOCs in agricultural practices. High costs associated with the process of formulation, mass production, registration, and marketing of synthetic VOCs can make their sale price prohibitive, and initial investments unproductive (Blum et al., 2011). Depending on the screening procedures, 5–10 years may be required for research and development of effective and specific synthetic blends of VOCs (Glare et al., 2012). Elevated production costs and scalability limitations have inevitably slowed down the use of VOCs in agriculture (Miresmailli and Isman, 2014). Besides, the management of VOC-based products in the field may become expensive, and require specific technical skills that usually growers and retailers do not possess. Nevertheless, the current increasing demand for eco-friendly and sustainable solutions to protect crops and enhance their productivity, also due to increasingly stringent limitations on chemical pesticides (see the European legislation on Plant Protection Products), could attract future investments making the use of VOCs in agriculture competitive, especially in conditions where VOC application can be controlled more effectively and problems with synthetic chemicals can manifest (e.g., greenhouses).

## Exploiting VOCs in Future Sustainable Agriculture: Outlook

### Reconsidering the Potential of VOC Applications in the Field

Over the last years, domestication and man-driven crop selection have mainly focused on high yields. However, this has at the same time selected against VOCs and other secondary metabolites that, in unstressed plants, may compete with primary production and reduce yields (Aharoni et al., 2004; Köllner et al., 2008; Klee and Tieman, 2013). Coping with fast climate change and heavier biotic and abiotic stress conditions now requires rediscovery and exploitation of the arsenal of natural defenses that has allowed wild crops to thrive in hostile, unfarmed conditions. This could be attained by selecting plants with different VOC emission blends to be used for novel intercropping schemes, or by co-cultivating modern crops with wild varieties to exploit VOC-driven plant-to-plant signaling and priming (Duhamel and Vandenkoornhuyse, 2013). If properly intercropped, stress-sensitive VOC-emitting plant species could act as “sentinel plants” and signal the occurrence of an imminent stressful situation to other neighboring crops of interest, which would be primed and become more resistant. Salt-stressed broad beans, wounded tobacco and pathogen-infected Arabidopsis have shown the ability to enhance resistance of neighboring unstressed plants through emission of VOCs (Dorokhov et al., 2012; Riedlmeier et al., 2017; Caparrotta et al., 2018). Alternatively, priming could be elicited by exposing plantlets to synthetic VOCs in confined environments (i.e., growth chamber or greenhouses) before transplanting in the field, thereby exploiting the long-term duration of the enhanced protection. Indeed, the time interval in which plants remain in the state of alertness (primed state) and therefore are more reactive to stress has demonstrated to last for days, weeks, or even be inherited through generations (Slaughter et al., 2012; Crisp et al., 2016). Modern crops could also be ‘rewildered’ (Palmgren et al., 2014) by directing breeding strategies toward the reintroduction of a more efficient capacity to produce and emit VOCs lost during domestication, but of use in plant protection. Recently developed techniques of genetic engineering (i.e., gene editing) could also be employed to restore desired wild/old traits into the plants of agricultural interest (Tieman et al., 2017). This could allow either ‘*de novo*’ emission of inducible VOCs (e.g., isoprenoids, Schnee et al., 2006), or modification of the blend already biosynthesized in leaves, such as GLVs (Shiojiri et al., 2006). However, difficulties may arise when engineering the biosynthesis of VOCs due to unexpected pleiotropic effects (Rosenkranz and Schnitzler, 2013).

### Novel Technologies for Employing VOCs in Smart Agriculture Practices

Nowadays, the availability of new analytical technologies such as high-resolution Proton Transfer Reaction “Time-of-Flight’ mass spectrometry (PTR-TOF-MS) makes possible instantaneous and highly sensitive detection of the whole spectra of VOCs with high resolving power (Graus et al., 2010). This can provide *in vivo* a complete and high-throughput measurement of the entire blend of VOCs (the “volatome”) emitted from plant leaves. Phenotyping the volatome could allow non-invasive screening of plant VOC profiles, assisting breeders in the selection of cultivars that successfully perform under changing environmental conditions and associated biotic stressors (Araus and Cairns, 2014). PTR-TOF-MS analysis could also enable a real-time diagnosis of the crop health status (Niederbacher et al., 2015), by monitoring in air the occurrence of specific VOC emissions (i.e., MeSA, sesquiterpenes) as stress biomarkers triggered by abiotic and biotic constraints (Karl et al., 2008; Chalal et al., 2015). Moreover, variations of VOC emission patterns over time can be used for precision agriculture purposes to monitor plant growth and development in the field. Likewise genomics and high throughput platforms for imaging and remote-sensing, real-time highly resolved VOC detection generate massive amount of data (Gandomi and Haider, 2015). This production of ‘big data’ requires computational analysis to extract patters and identify features useful for phenotyping (Singh et al., 2016). Implementation of machine learning tools to process information on VOC emissions along with environmental parameters collected in the field by multiple sensors will allow exploration of big data in order to measure plant performance and recognize early symptoms of stress.

## Author Contributions

FB and IB conceived and wrote the manuscript. FL critically revised the content and contributed to the writing.

### Conflict of Interest Statement

The authors declare that the research was conducted in the absence of any commercial or financial relationships that could be construed as a potential conflict of interest.
